# Cardiovascular Risk Factors in Middle-Aged Lithuanian Men: A Comparative Study of an Apparently Resistant Hypertension Group

**DOI:** 10.3390/biomedicines13020435

**Published:** 2025-02-11

**Authors:** Vaida Šileikienė, Vilma Dženkevičiūtė, Alma Čypienė, Martynas Bublys, Roma Puronaitė, Jolita Badarienė, Aleksandras Laucevičius, Eglė Butkevičiūtė, Egidija Rinkūnienė

**Affiliations:** 1 Clinic of Cardiac and Vascular Diseases, Santariškių Str. 2, 08406 Vilnius, Lithuania; sileikiene.vaida@gmail.com (V.Š.); vilma.dzenkeviviute@santa.lt (V.D.); alma.cypiene@santa.lt (A.Č.); jolita.badariene@santa.lt (J.B.); aleksandras.laucevicius@santa.lt (A.L.); egidija.rinkuniene@santa.lt (E.R.); 2 Faculty of Medicine, Vilnius University, Ciurlionio Str. 21, 03101 Vilnius, Lithuania; 3 Research Institute Centre for Innovative Medicine, Santariskiu Str. 5, 08406 Vilnius, Lithuania; 4 Institute of Data Science and Digital Technologies, Faculty of Mathematics and Informatics, Vilnius University, Akademijos 4, 08412 Vilnius, Lithuania; 5 Department of Software Engineering, Faculty of Informatics, Kaunas University of Technology, Studentu Str. 50, 51368 Kaunas, Lithuania; egle.butkeviciute@ktu.lt

**Keywords:** cardiovascular risk factors, prevalence, apparently resistant hypertension, hypertension, management

## Abstract

**Background/Objectives**: Hypertension (HTN) is a significant risk factor for cardiovascular disease (CVD), and a subset of patients exhibits apparently resistant hypertension (aRHTN), where blood pressure remains inadequately controlled despite treatment. This study aims to assess the prevalence of cardiovascular risk factors in middle-aged Lithuanian men with HTN and aRHTN, as well as to evaluate the effectiveness of hypertension management in these groups. **Methods**: Data from 52,012 men participating in the Lithuanian High Cardiovascular Risk Programme (LitHiR) between 2009 and 2019 were analysed. Participants were categorised into two groups: treated hypertension (HTN) and apparent resistant hypertension (aRHTN). Despite treatment, the aRHTN group included those who failed to achieve their target blood pressure. The prevalence of cardiovascular risk factors (dyslipidaemia, diabetes, metabolic syndrome, obesity, physical inactivity, and an unbalanced diet) was compared between the groups. **Results**: The overall prevalence of HTN was 47%, with 9.9% of treated hypertensive men having aRHTN. Dyslipidemia was both groups’ most prevalent risk factor (94.1% in HTN vs. 95.5% in aRHTN, *p* < 0.001). Men with aRHTN exhibited higher rates of diabetes (25.9% vs. 18.5%, *p* < 0.001), metabolic syndrome (75.3% vs. 66.3%, *p* < 0.001), and left ventricular hypertrophy (59.4% vs. 43.1%, *p* < 0.001). Treatment success was significantly lower in the aRHTN group (7.57% vs. 28.4%, *p* < 0.001). **Conclusions**: Hypertension affects almost half of the studied population, with 10% of treated hypertensives exhibiting aRHTN. The aRHTN group had a higher number of additional cardiovascular risk factors and lower treatment success rates. Improved management of cardiovascular risk factors is crucial, especially in the aRHTN population, to reduce the burden of CVD.

## 1. Introduction

Hypertension (>140/90 mmHg) is one of the leading risk factors for death and disability worldwide, predominantly affecting low- and middle-income countries. The global burden of hypertension has steadily increased over time. Between 1990 and 2019, the number of individuals living with hypertension doubled, rising from 650 million to 1.3 billion worldwide. This ongoing rise in hypertension prevalence is primarily attributed to population growth, lifestyle changes, and an ageing population [1]. At present, hypertension is responsible for over 9 million deaths each year and contributes to 9% of global disability-adjusted life years [2]. The prevalence of hypertension in Lithuania reflects the global trend and remains elevated, rising to 345 cases per 1000 people in 2022 [3,4].

Resistant hypertension is a serious form of high blood pressure that was recognised more than 50 years ago as the inability to sustain normal blood pressure despite receiving medication [5]. The European Society of Hypertension (ESH) and the European Society of Cardiology (ESC) established the groundwork for the further categorisation and advancement of research on resistant arterial hypertension by introducing an improved definition in 2007, which states that blood pressure cannot be reduced after the prescription of at least three medications (including a diuretic) at appropriate doses [6,7]. This definition provided clear inclusion criteria but was not specific enough to identify cases of resistant hypertension and represented a heterogeneous population with markedly different disease risks. After collecting more concrete data on the factors responsible for the misdiagnosis of resistant arterial hypertension, the following leading causes were identified: white coat hypertension, inaccurate blood pressure measurement in the office, low adherence to prescribed treatment, and clinical inertia [5,8,9,10]. Following this new research, the ESH/ESC guidelines now recommend additional diagnostic measures such as ambulatory blood pressure measurement, confirmation of treatment adherence, and exclusion of secondary causes of hypertension to accurately diagnose true resistant hypertension [11]. If the criteria below are not met, hypertension should be classified as apparently treatment-resistant hypertension [7]. According to current diagnostic algorithms, researchers discovered that the prevalence of resistant hypertension is 10.3% among all individuals treated with antihypertensives, while apparently treatment-resistant hypertension represents 14.7% of those studied [12]. The prevalence of true resistant hypertension is even greater in particular patient groups, such as those with chronic kidney disease, kidney transplant recipients, and elderly individuals (22.9%, 56%, and 12.3%, respectively) [12]. Resistant hypertension has a relatively poor prognosis. J. Sim et al. found that all-cause mortality is significantly higher in individuals with resistant hypertension compared to those with non-resistant hypertension. In assessing the management outcomes of resistant hypertension, the risk of developing end-stage renal disease and experiencing a cerebrovascular event was 25% and 23% higher, respectively, in uncontrolled resistant hypertension than in controlled resistant hypertension, according to a retrospective cohort study conducted over five years [13]. Resistant hypertension is a complex diagnostic condition with a poor prognosis for overall mortality. It is linked to additional risk factors that promote cardiovascular disease, such as elevated BMI, waist circumference, diabetes, and left ventricular hypertrophy [14].

This study aimed to determine the prevalence of cardiovascular risk factors, such as diabetes mellitus, smoking, a family history of coronary heart disease, dyslipidaemia, physical activity, diet, ECG abnormalities, and metabolic syndrome, among middle-aged Lithuanian men. These men were categorised into groups of hypertension or apparently treatment-resistant hypertension.

## 2. Materials and Methods

### 2.1. Data Source

The Lithuanian High Cardiovascular Risk Programme (LitHiR) is a primary prevention initiative for middle-aged men and women that seeks to identify patients at high risk of cardiovascular disease and implement preventative strategies [15]. Launched in 2006, LitHiR was developed into a nationwide patient care programme, with 91.6% of primary health care institutions (PHCIs) in Lithuania participating by 2016. The target population consisted of men (ages 40–54) and women (ages 50–64) without evident cardiovascular disease who underwent a comprehensive physical examination, lifestyle assessment, and coronary risk analysis (including smoking, physical activity, dietary habits, and family history of coronary heart disease), along with anthropometric measurements. Laboratory tests were conducted to assess the lipid profile and fasting glycaemia. A 12-lead ECG was recorded to ascertain left ventricular hypertrophy [16].

### 2.2. Participants

This report presents data from 52,012 men participating in the LitHiR programme from 2009 to 2019. Based on their hypertension status, participants were categorised into five groups.

Individuals with normal blood pressure (BP) who were not taking antihypertensive medication were classified as normotensive. Participants not prescribed medication but with a prior hypertension diagnosis or an abnormal BP measurement during the PHCI examination were divided into two groups: untreated previously diagnosed hypertension (UPDH) and untreated recently diagnosed hypertension (URDH). The treated hypertension group (HTN) comprised individuals diagnosed with hypertension who had commenced treatment. Participants in the apparently treatment-resistant hypertension group (aRHTN) had been diagnosed with primary hypertension. They had initiated treatment that proved ineffective in lowering blood pressure (<140 mmHg and/or <90 mmHg) despite being prescribed three antihypertensives at appropriate doses, including a diuretic, or were managed with four or more antihypertensives irrespective of blood pressure levels. The treatment of hypertension and apparently resistant hypertension (aRHTN) was considered successful if the patient had undergone appropriate treatment and their blood pressure was within the normal range at the time of the medical examination. The diagnostic criteria for aRHTN have been established based on the 2007 European Society of Cardiology/European Society of Hypertension (ESC/ESH) guidelines for the management of arterial hypertension.

### 2.3. Data Collection

The BMI was calculated using the following formula: $\frac{weight\left(kg\right)}{{height}^{2}\left(m^{2}\right)}$. Participants with a BMI below 18.5 kg/m^2^ were considered to have low weight, while those with a BMI of 18.5–24.99 kg/m^2^ were classified as having normal weight. A BMI of 25.0–29.99 kg/m^2^ indicated overweight, 30.0–34.99 kg/m^2^ denoted grade 1 obesity, 35.0–39.99 kg/m^2^ denoted grade 2 obesity, and a BMI of 40 kg/m^2^ or more was categorised as grade 3 obesity. Blood pressure was measured while seated after at least five minutes of rest. A total of three measurements were taken, and the average was calculated. The dominant arm was positioned at heart level, and appropriate cuffs were used. All blood pressure measurements were conducted at PHCI. Insufficient physical activity was defined as failing to exercise at least three times per week for 45 min. An unbalanced diet was regarded as present if the participants’ daily nutrition included a high proportion of animal fats, sugar, salt, and/or a low proportion of plant-based foods. Assessments of physical activity, diet, and smoking habits relied solely on self-reported data, without implementing objective tests to reduce positive bias. Information regarding diabetes mellitus status (Type I or II) was gathered from medical histories, and all participants’ fasting glucose concentrations were measured. Serum total cholesterol (TC), high-density lipoprotein cholesterol (HDL-C), and triglycerides were evaluated, with dyslipidaemia diagnosed based on the European Guidelines for the Prevention of CVD if any of the following criteria were met: TC > 5 mmol/L, LDL-C > 3 mmol/L, HDL < 1.0 mmol/L, or TGs > 1.7 mmol/L [17]. Genetic dyslipidaemias were excluded from our analysis. Metabolic syndrome was diagnosed with the presence of at least three of five risk factors according to the modified National Cholesterol Education Programme III criteria (increased waist circumference > 102 cm in men, triglycerides > 1.7 mmol/L, HDL cholesterol < 1.03 mmol/L, systolic blood pressure ≥ 130 mmHg or diastolic blood pressure ≥ 85 mmHg or the patient is taking antihypertensives, fasting glucose ≥ 5.6 mmol/L). Waist circumference (WC) was measured just above the iliac crest with a centimetre tape while the patient stood and breathed steadily and normally [18].

The study protocol was approved by the Vilnius Regional Ethics Committee for Biomedical Research (No. 158200-15-816-329). It was impossible to obtain written informed consent from each patient, as stated in our study protocol submitted to the Regional Biomedical Research Ethics Committee.

### 2.4. Statistical Analysis

Statistical data analysis was conducted using R version 4.4.1 and Excel software. Continuous variables were reported as means ± standard deviations (SDs), while categorical variables were expressed as absolute frequencies (*n*) and percentages (%) of the sample. Normality was assessed using histograms, and for large sample sizes, normality was assumed based on the central limit theorem. The two groups were compared using either the *t*-test or Mann–Whitney U-test for continuous variables, and Pearson’s chi-square test or Fisher’s exact test for categorical variables. Statistical significance was established at *p* < 0.05.

## 3. Results

### 3.1. General Characteristics

**Figure 1:**
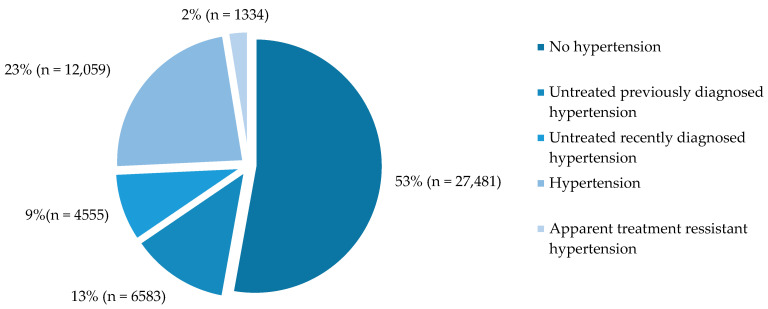
Hypertension prevalence in analysed population.

Data were collected from 52,012 middle-aged male participants. Among this large cohort, 47% (*n* = 24,531) were diagnosed with arterial hypertension, and only 23% (*n* = 12,059) of men received treatment. In total, data from 13,393 men who met the criteria for treated hypertension and apparently resistant hypertension were analysed further. The actual prevalence of aRHTN in the treated hypertensive population was 9.9% (Figure 1).

The average age, BMI, and waist circumference were higher in the group with apparently treatment-resistant hypertension. The values for total cholesterol and low-density lipoprotein cholesterol did not differ significantly; additional study characteristics are presented in Table 1.

Dyslipidaemia was the most common cardiovascular risk factor in both the treated and apparently resistant hypertension groups (94.1% and 95.5%, respectively). Men in the apparently resistant hypertension group had a significantly higher prevalence of history of diabetes (*p* < 0.001), metabolic syndrome (*p* < 0.001), left ventricular hypertrophy (*p* < 0.001), unbalanced diet (*p* < 0.001), and insufficient physical activity (*p* < 0.001). The reduced HDL-C concentration was insignificantly higher in the HTN group (*p* = 0.903), but the increased TG concentration was notable in the aRHTN group (*p* < 0.001). Only smoking and a family history of premature CHD were insignificantly more common in the HTN group (Table 2).

### 3.2. BMI Group Analysis in a Hypertensive Population

**Figure 2:**
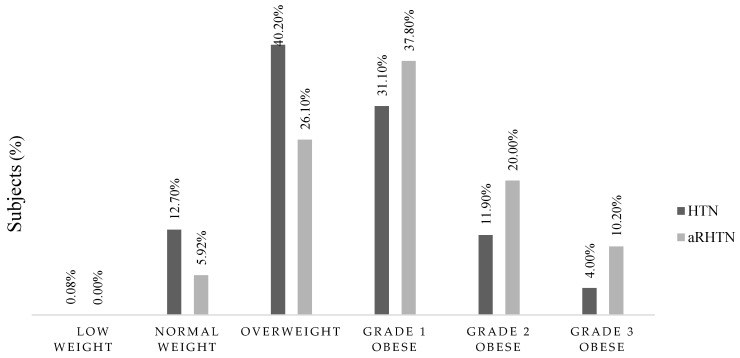
BMI distribution in HTN and aRHTN groups.

Divided into BMI categories, the apparently resistant hypertensive group had twice as many men classified as obese grade 2 and 3, while the number of overweight individuals was significantly higher in the hypertensive group. Overall, the BMI category for underweight men was the least pronounced (Figure 2).

### 3.3. Hypertension Treatment Control and Cardiovascular Risk

The group of apparently treatment-resistant hypertensives exhibited a significantly lower rate of successfully treated hypertension compared to the general hypertensive population (7.57% (*n* = 101) vs. 28.4% (*n* = 3426); *p* < 0.001). The analysis of cardiovascular risk factors between successfully and unsuccessfully treated aRHTN groups revealed that 11 out of 12 risk factors analysed showed no statistically significant differences. Only the increased LDL-C concentration occurred more frequently in the group of unsuccessfully treated patients compared to the group of successfully treated aRHTN patients (Table 3). Among the analysed hypertensive population, all cardiovascular risk factors were more prevalent in the group with inadequate blood pressure reduction. Smoking (36% (*n* = 3556) vs. 30.4% (*n* = 1073); *p* < 0.001), increased BMI (52.1% (*n* = 5140) vs. 40.8% (*n* = 1440); *p* < 0.001), insufficient physical activity (57.8% (*n* = 5703) vs. 53.4% (*n* = 1882); *p* < 0.001), an unbalanced diet (71% (*n* = 7002) vs. 63.2% (*n* = 2229); *p* < 0.001), ECG changes (47.5% (*n* = 4686) vs. 37.1% (*n* = 1308); *p* < 0.001), and metabolic syndrome (68% (*n* = 6711) vs. 64.9% (*n* = 2290); *p* < 0.001) were strongly associated with inadequate blood pressure control. In contrast, the differences between the groups were less pronounced for diabetes, dyslipidaemia, and elevated LDL-C concentration (Table 4).

## 4. Discussion

Pooled data from 2009 to 2019 indicated that the prevalence of seemingly treatment-resistant hypertension among treated hypertensive men was 9.9%. A recent meta-analysis assessed 3.2 million patients. According to the data, the rate of apparently resistant hypertension was 14.7% [12]. In the American population, the prevalence of aRHTN was somewhat higher, accounting for 17.7% of those treated for hypertension [19]. This high prevalence of ostensibly treatment-resistant hypertension may stem from the difficulty in differentiating aRHTN from other subtypes of hypertension. True resistant hypertension is diagnosed less frequently than aRHTN. In a descriptive cross-sectional study conducted in China, the prevalence of true resistant arterial hypertension was 7.43% of the treated population [20]. Similar data were presented in a UK cohort study, which found that the prevalence of true RHT was less than 6.5% of treated hypertensives [21]. Our study was limited to identifying aRHTN, which is not the most specific cluster for resistant hypertension. This meant that the comparison between our groups of hypertensives and aRHTN was compromised by analysing rather similar datasets that tended to overlap. HTN and true resistant hypertension populations may have an even more distinct distribution of cardiovascular risk factors [14].

Dyslipidaemia was the most prevalent cardiovascular risk factor in our study, affecting both the hypertensive patient group and the group of apparently resistant hypertensive patients. The prevalence of elevated LDL-C concentration alone was 77.3% in the HTN group and 79.3% in the aRHTN group. Hypertension and dyslipidaemia are independent cardiovascular risk factors that contribute to extensive arterial wall remodelling, promoting oxidative stress and subendothelial lipid accumulation. This results in a more rapid development of atherosclerosis and hypertension-mediated organ damage (HMOD) [22]. Kutkiene et al. concluded that the presence of dyslipidaemia significantly raises the incidence of arterial hypertension, diabetes, and metabolic syndrome in the studied population [23]. We found no statistically significant difference in the prevalence of dyslipidaemia between the HTN and aRHTN groups. However, a statistically significant difference emerged when analysing the prevalence of abnormal LDL-C concentrations in the groups with successfully treated aRHTN and resistant hypertension (69.3% and 80.1%, respectively; *p* = 0.014). Similar results were reported in a large Spanish cohort study. De la Sierra et al. discovered that individuals with controlled hypertension had significantly fewer cardiovascular risk factors (diabetes, dyslipidaemia, impaired renal function, microalbuminuria, left ventricular hypertrophy) than those in the treated resistant hypertension group [24]. Increased BMI, diabetes, and metabolic syndrome were more prevalent in individuals with apparently treatment-resistant hypertension than in the hypertensive group. Oliveras et al. published a meta-analysis with similar findings. All groups with resistant hypertension exhibited a higher prevalence of BMI and diabetes compared to those with controlled hypertension. The prevalence of metabolic syndrome was nearly twice as high in the resistant hypertension group as in the controlled hypertension group (71.1% vs. 39.5%; *p* <0.001) [25].

Our study was designed to categorise participants as physically inactive if the men analysed exercised fewer than three times a week for 40 min. Insufficient physical activity was significantly more prevalent in the aRHTN group at 63.6%. Similar results were reported in the REGARDS study, where the prevalence of unhealthy lifestyle factors in the aRHTN group exceeded 55% [26]. Ozemek et al. found that 90 to 150 min per week of aerobic physical activity at a heart rate reserve of 65 to 75 can lower systolic blood pressure by 5 to 8 mmHg in hypertensive patients [27]. Another meta-analysis of three randomised controlled trials (*n* = 144) demonstrated that regular exercise training (8 to 12 weeks, three sessions per week) significantly reduced 24 h blood pressure (−9.9 mmHg for systolic blood pressure and −5 mmHg for diastolic blood pressure) as well as daytime ambulatory blood pressure (−11.7 mmHg for systolic blood pressure and −7.4 mmHg for diastolic blood pressure) [28]. However, in our study, no differences in blood pressure were found between the HTN and aRHTN groups, although a significant difference in the prevalence of insufficient physical activity was noted. An unbalanced diet was also statistically more prevalent in the aRHTN group. The recent TRIUMPH randomised clinical trial produced similar results. A centre-based lifestyle intervention (C-LIFE group), which included exercise training, a sodium- and calorie-restricted diet (DASH diet plan), and weight management, was compared with standardised education and physician advice (SEPA group) for the treatment of patients with resistant hypertension. The C-LIFE group experienced a greater reduction in systolic blood pressure in the clinic compared to the SEPA group (–12.5 mmHg vs. –7.1 mmHg; *p* = 0.05), and the decrease in systolic blood pressure in the 24 h ambulatory setting was even more pronounced (–7.0 mmHg vs. –0.3 mmHg; *p* = 0.001) [29]. Additionally, another meta-analysis of randomised controlled trials indicated that the DASH diet (which reduced animal fats and sodium while increasing plant-based foods) lowered both sBP and dBP compared to a control diet (difference in mean values: −3.2 mm Hg; *p* < 0.001 and −2.5 mm Hg; *p* < 0.001) [30].

## 5. Limitations

This study focuses exclusively on men aged 40 to 55, as the LitHiR primary prevention programme targets this age group due to their heightened risk of developing cardiovascular disease. Furthermore, this study did not consider psychosocial, socioeconomic, and other risk factors, nor was it an objective assessment of physical activity, diet, and smoking habits, as these relied solely on self-reported data. Without objective measurements of these behaviours (for example, using physical activity trackers or dietary questionnaires), verifying the accuracy of the self-reported data is challenging, limiting the validity of the study’s conclusions. Consequently, caution should be exercised when interpreting the association between self-reported lifestyle factors and cardiovascular health in this context sample.

## 6. Conclusions

Nearly half of the studied population was diagnosed with hypertension, and almost one in ten treated hypertensive men had apparently resistant hypertension. The most prevalent cardiovascular risk factors within the aRHTN and hypertensive groups were dyslipidaemia and elevated LDL-C levels. Nevertheless, other cardiovascular risk factors, such as diabetes, BMI (>30 kg/m^2^), insufficient physical activity, unbalanced diet, metabolic syndrome, and ECG changes, were more frequently observed in the aRHTN group. Participants with treated hypertension demonstrated significantly better treatment success compared to those with apparently resistant hypertension. In men with hypertension, greater treatment success was linked to improved cardiovascular risk management. Conversely, inadequate blood pressure control was strongly correlated with a higher prevalence of risk factors, including smoking, increased BMI, insufficient physical activity, unbalanced diet, ECG changes, and metabolic syndrome. However, conditions such as diabetes, dyslipidaemia, and a family history of premature CHD showed weaker or no significant associations.

## Figures and Tables

**Table 1:** General characteristics of the study sample.

	HTN (*n* = 12,059)	aRHTN (*n* = 1334)	HTN vs. aRHTN
	Mean ± SD	Mean ± SD	*p*
Age (years)	47.7 ± 4.34	48.4 ± 4.19	<0.001
BMI (kg/m^2^)	30.3 ± 5.07	32.8 ± 5.57	<0.001
WC (cm)	103 ± 12.9	109 ± 13.3	<0.001
sBP (mmHg)	143 ± 15.7	151 ± 17.1	<0.001
dBP (mmHg)	88.2 ± 10.0	92.4 ± 10.4	<0.001
HR (bpm)	73.0 ± 9.29	73.9 ± 9.39	0.007
Fasting glycemia (mmol/L)	5.83 ± 1.61	6.12 ± 1.74	<0.001
TC (mmol/L)	5.93 ± 1.24	5.98 ± 1.22	0.713
LDL-C (mmol/L)	3.77 ± 1.06	3.80 ± 1.05	0.848
HDL-C (mmol/L)	1.33 ± 0.42	1.26 ± 0.36	<0.001
TGs (mmol/L)	2.04 ± 1.64	2.32 ± 1.82	<0.001

Abbreviations: HTN—hypertension; aRHTN—apparently treatment-resistant hypertension; BMI—body mass index; WC—waist circumference; sBP—systolic blood pressure; dBP—diastolic blood pressure; HR—heart rate; TC—total cholesterol; LDL-C—low-density lipoprotein cholesterol; HDL-C—high-density lipoprotein cholesterol; TGs—triglycerides.

**Table 2:** Prevalence of cardiovascular risk factors in hypertensive and aRHTN groups.

	HTN (*n* = 12,059)	aRHTN (*n* = 1334)	HTN vs. aRHTN
	Number (%)	Number (%)	*p*
DM	2227 (18.5%)	346 (25.9%)	
Smoking	4187 (34.7%)	442 (33.1%)	0.26
Family History of Premature CHD	3618 (30.0%)	399 (29.9%)	0.878
BMI > 30 kg/m^2^	5673 (47.0%)	907 (68.0%)	<0.001
Dyslipidaemia	11,348 (94.1%)	1274 (95.5%)	0.048
Insufficient physical activity	6736 (55.9%)	849 (63.6%)	<0.001
Unbalanced nutrition (Diet)	8232 (68.3%)	999 (74.9%)	<0.001
ECG changes: LV Hypertrophy	5202 (43.1%)	792 (59.4%)	<0.001
Metabolic syndrome (MS)	7996 (66.3%)	1005 (75.3%)	<0.001
HDL-C < 1.03 mmol/L	5477 (45.4%)	603 (45.2%)	0.903
TGs > 1.7 mmol/L	7378 (61.2%)	889 (66.6%)	<0.001
LDL-C (>3 mml/L)	9319 (77.3%)	1058 (79.3%)	0.134
Successful HTN control	3426 (28.4%)	101 (7.57%)	<0.001

Abbreviations: DM—Diabetes mellitus; CHD—coronary heart disease; BMI—body mass index; LV—left ventricle; MS—Metabolic syndrome; LDL-C—low-density lipoprotein cholesterol; TGs—triglycerides.

**Table 3:** Cardiovascular risk factors and aRHTN management.

	Failed (*n* = 1233)	Achieved (*n* = 101)	Failed vs. Achieved
	Number (%)	Number (%)	*p*
DM (I or II) anamnesis	318 (25.8%)	28 (27.7%)	0.758
Smoking	409 (33.2%)	33 (32.7%)	1
Family history of premature CHD	369 (29.9%)	30 (29.7%)	0.957
BMI > 30 kg/m^2^	842 (68.3%)	65 (64.4%)	0.482
Dyslipidaemia	1178 (95.5%)	96 (95%)	0.801
Insufficient physical activity	785 (63.7%)	64 (63.4%)	1
Unbalanced nutrition (Diet)	927 (75.2%)	72 (71.3%)	0.454
ECG changes: LV Hypertrophy	739 (59.9%)	53 (52.5%)	0.173
Metabolic syndrome (MS)	548 (44.4%)	80 (79.2%)	0.413
MS (reduced HDL-C)	5477 (45.4%)	55 (54.5%)	0.066
MS (increased TGs)	822 (66.7%)	67 (66.3%)	1
LDL-C (> 3 mml/L)	988 (80.1%)	70 (69.3%)	0.014

Abbreviations: DM—Diabetes mellitus; CHD—coronary heart disease; BMI—body mass index; LV—left ventricle; MS—Metabolic syndrome; LDL-C—low-density lipoprotein cholesterol; TGs—triglycerides.

**Table 4:** Cardiovascular risk factors and hypertension management.

	Failed (*n* = 9866)	Achieved (*n* = 3527)	Failed vs. Achieved
	Number (%)	Number (%)	*p*
DM	1939 (19.7%)	634 (18.0%)	0.032
Smoking	3556 (36.0%)	1073 (30.4%)	<0.001
Family history of premature CHD	1685 (17.1%)	591 (16.8%)	0.239
BMI > 30 kg/m^2^	5140 (52.1%)	1440 (40.8%)	<0.001
Dyslipidaemia	9325 (94.5%)	3297 (93.5%)	0.026
Insufficient physical activity	5703 (57.8%)	1882 (53.4%)	<0.001
Unbalanced nutrition (Diet)	7002 (71.0%)	2229 (63.2%)	<0.001
ECG changes: LV Hypertrophy	4686 (47.5%)	1308 (37.1%)	<0.001
Metabolic syndrome (MS)	6711 (68.0%)	2290 (64.9%)	0.001
HDL-C < 1.03 mmol/L	4384 (44.4%)	1696 (48.1%)	<0.001
TGs > 1.7 mmol/L	6143 (62.3%)	2124 (60.2%)	0.034
LDL-C (>3 mml/L)	7692 (78.0%)	2685 (76.1%)	0.026

Abbreviations: DM—Diabetes mellitus; CHD—coronary heart disease; BMI—body mass index; LV—left ventricle; MS—Metabolic syndrome; LDL-C—low-density lipoprotein cholesterol; TGs—triglycerides.

## Data Availability

The data presented in this study is available on request from the corresponding author. The data is not publicly available due to privacy and ethical restrictions.

## Abbreviations

The following abbreviations are used in this manuscript:

aRHTN	Apparently resistant hypertension
HTN	Hypertension
LitHiR	The Lithuanian High Cardiovascular Risk Program
DM	Diabetes mellitus
CHD	Coronary heart disease
BMI	Body mass index
LV	Left ventricle
MS	Metabolic syndrome
HDL-C	High-density lipoprotein cholesterol
LDL-C	Low-density lipoprotein cholesterol
TGs	Triglycerides
WC	Waist circumference
sBP	Systolic blood pressure
dBP	Diastolic blood pressure
HR	Heart rate
